# An Involuntary Proving of Lac Caninum

**Published:** 1888-09

**Authors:** H. C. Morrow


### AN INVOLUNTARY PROVING OF LAC CANINUM.

Mrs. F., a widow, small, petite, about thirty years of age, sought relief for hay-fever, some of the premonitory symptoms of which were commencing to develop. Lac caninum covered all her symptoms, and one dose of the CM (Swan) was administered dry on the tongue with a plentiful supply of Sac. Lac.

Took powder in morning. In evening of same day eyes commenced to pain, and had a confused feeling in the head. Awoke at four A. M. next morning with rawness and soreness in throat and aching in lower extremities.

The following is a recapitulation of her symptoms from first to last according to the Hahnemannian schema:

Irritable and cross, wanted to "pulverize" her physician for making her sick. Headache commencing in occiput (third day) and extending to vertex and forehead; throbbing in forehead and temples, finally settling in eyes; eyes very sensitive to light during headache; eye-balls sore and painful, pains extending deep back into the brain with the headache. Headache commencing all over head at once (second day) in the morning, a dull, heavy, confused feeling in head all day, becoming a severe ache toward evening, and settling in eyes and temples. Headache worse from motion and stooping; better from cold applications. Swelling and inflammation of upper and lower lids of both eyes, the left the worst. Tired feeling in eyes, considerable lachrymation—both eyes—redness of conjunctiva, eye-ball sore, aching and painful; pains extending deep back into head, with headache; pains in eyes better from cold (wet) applications; eyes sensitive to artificial light; discharge of clear white mucus from nose and sneezing; earache the first time in her life on right side; aching deep in the ear, worse from cold air; had to keep ear covered all the time; pain in ear better by pressure with the point of the finger in meatus; external ear sore and painful; soreness and swelling on right side from ear down side of face in the region of parotid gland and angle of lower jaw; swelling and soreness of glands at angle of lower jaw, right side; soreness extending from right ear down side of neck to right shoulder, painful when turning the head; gums, upper and lower, very sore and red; tongue coated white, breath offensive; sensation of a hair in back part of mouth on right side, which she tried ineffectually to wipe away; profuse expectoration of saliva.

Awoke about four A. M. (second day) with a feeling that she was going to have sore throat; rawness and soreness in throat on right side. Swollen feeling in throat. Feeling as if she wanted to expectorate but could get nothing up. Sensation of a sac (lump?) in her throat on right side which seemed to descend when she swallowed, and scraped or rubbed against the mucous membrane as it went down; returning after deglutition.

Fauces and tonsils very sore and red. Right tonsil appeared puckered and drawn up from circumference to centre.

In centre of right tonsil, a small *black spot* about the size of a pin-head. Two long shaped ulcers on right tonsil, toward the inner edge. On third day a ring of small yellow blisters around each ulcer, which later presented appearance of a false membrane. False membrane on right tonsil but not on left. Left tonsil became sore and inflamed on second day, when the right was not nearly so painful; by evening pain and soreness returned to right side when the left side was relieved. This alternation from side to side continued one week when the painful symptoms wholly subsided.

All deglutition painful, but worse when swallowing solid food. Pain extends to ear when swallowing. Pains in throat worse in cold air.

No appetite for anything.

Cramping pain in calf of right leg at night. Was compelled to get up and rub it, which relieved. Had this cramping every night for a week. A knot (objective subjective) in calf of right leg when cramping; sensation as if some one had a stick in muscles and was twisting it around. (Never had cramp except when pregnant several years before.) During the whole proving feverish in the evenings. Flashes of heat, commencing in chest and extending up over face and head, when she would break out in sweat, which would dry up in a short time; face would become moist first and become dry first. Sensation over whole chest as if dripping with sweat when only slightly moist.

Sleep restless, tossing about. Horrible dreams.

The time from the first feeling of illness until all painful symptoms subsided was one week. With the exception of the sneezing, for which I prescribed, she felt perfectly well. Had not had but one attack of sore throat during the whole winter. It was a Lac caninum sore throat, though not nearly so severe as the one produced by the drug, considering that it came on in only a few hours, the one dose and the potency.

I consider it a good proving. By comparison with Swan's *Mat. Med.*, it will be seen that some of the symptoms have never been produced in any previous proving.

H. C. Morrow, M. D.

